# Narrow-band high-lying excitons with negative-mass electrons in monolayer WSe_2_

**DOI:** 10.1038/s41467-021-25499-2

**Published:** 2021-09-17

**Authors:** Kai-Qiang Lin, Chin Shen Ong, Sebastian Bange, Paulo E. Faria Junior, Bo Peng, Jonas D. Ziegler, Jonas Zipfel, Christian Bäuml, Nicola Paradiso, Kenji Watanabe, Takashi Taniguchi, Christoph Strunk, Bartomeu Monserrat, Jaroslav Fabian, Alexey Chernikov, Diana Y. Qiu, Steven G. Louie, John M. Lupton

**Affiliations:** 1 Department of Physics, University of Regensburg, Regensburg, Germany; 2 Department of Physics, University of California at Berkeley, Berkeley, CA USA; 3 Materials Sciences Division, Lawrence Berkeley National Laboratory, Berkeley, CA USA; 4 Cavendish Laboratory, University of Cambridge, Cambridge, UK; 5 Research Center for Functional Materials, National Institute for Materials Science, Tsukuba, Japan; 6 International Center for Materials Nanoarchitectonics, National Institute for Materials Science, Tsukuba, Japan; 7 Department of Materials Science and Metallurgy, University of Cambridge, Cambridge, UK; 8 Dresden Integrated Center for Applied Physics and Photonic Materials (IAPP) and Würzburg-Dresden Cluster of Excellence ct.qmat, Technische Universität Dresden, Dresden, Germany; 9 Department of Mechanical Engineering and Materials Science, Yale University, Yale, CT USA

**Keywords:** Two-dimensional materials, Quantum optics

## Abstract

Monolayer transition-metal dichalcogenides (TMDCs) show a wealth of exciton physics. Here, we report the existence of a new excitonic species, the high-lying exciton (HX), in single-layer WSe_2_ with an energy of ~3.4 eV, almost twice the band-edge A-exciton energy, with a linewidth as narrow as 5.8 meV. The HX is populated through momentum-selective optical excitation in the *K*-valleys and is identified in upconverted photoluminescence (UPL) in the UV spectral region. Strong electron-phonon coupling results in a cascaded phonon progression with equidistant peaks in the luminescence spectrum, resolvable to ninth order. *Ab initio GW*-BSE calculations with full electron-hole correlations explain HX formation and unmask the admixture of upper conduction-band states to this complex many-body excitation. These calculations suggest that the HX is comprised of electrons of negative mass. The coincidence of such high-lying excitonic species at around twice the energy of band-edge excitons rationalizes the excitonic quantum-interference phenomenon recently discovered in optical second-harmonic generation (SHG) and explains the efficient Auger-like annihilation of band-edge excitons.

## Abstract (web summary)

Here, the authors report on evidence of an excitonic species formed by electrons in high-energy conduction band states with a negative effective mass, explaining previous observations of quantum interference phenomena in two-dimensional semiconductors.

## Introduction

Optical transitions in atoms occur between different discrete electronic states. Coherent coupling can arise between transition pathways, giving rise to quantum-interference phenomena such as electromagnetically induced transparency (EIT)^1,2^. Analogous multilevel systems in semiconductor quantum wells—intersubband transitions—have opened up applications in the infrared and terahertz frequency range^3^. Further extension of such phenomena towards the visible spectral range is desirable, particularly in semiconductors that are promising for optoelectronic applications. In a semiconductor, at sufficiently low temperatures, an electron in the conduction band and a hole in the valence band can interact with each other to form a correlated state—an exciton. In monolayer TMDCs, excitons can exhibit large optical transition strengths, giving rise to radiative lifetimes on the order of 150 fs^4^—over 10,000 times shorter than those of atoms. These band-edge excitons can therefore interact strongly with incident radiation^5,6^. Promoting excitons into states at significantly higher energies—beyond their continuum limit—involves exciting the electron into high-lying conduction bands. If such transitions in a crystal occur vertically in momentum space, high-energy excitons would contribute to a multilevel excitonic structure that could support atom-like quantum interference. The common expectation would suggest that these excitons are resonant states in the two-particle continuum that relax quickly towards the optical band gap and therefore do not have appreciable coherence time. Recently, however, clear signatures of excitonic quantum interference have been observed in monolayer WSe_2_ when driven by a 140 fs laser pulse^7^. In addition, efficient Auger-like upconversion of luminescence in monolayer WSe_2_ has been attributed to electronic transitions to upper conduction bands^8^. These experiments suggest that the optically bright band-edge exciton can couple to a discrete metastable state of approximately twice the energy, located in the same region of momentum space. More surprisingly, the coherence time of such a metastable state was inferred from the excitonic quantum-interference experiments to be as long as that of the band-edge A-exciton^7,9^. If correct, monolayer WSe_2_ would comprise a multilevel excitonic energy structure reminiscent of that of dilute atomic-gas systems^1,2^.

Here, we probe this HX directly by upconverted photoluminescence (UPL) in the UV and identify its origin by ab initio *GW*-BSE calculations. The HX appears at an energy close to twice that of the band-edge exciton and features a linewidth as narrow as 5.8 meV, consistent with a coherence time of at least 100 fs as is necessary for the observed excitonic quantum interference phenomenon^7,10^. Such an exciton appears particularly unexpected when the independent-particle picture of interband transitions suggests that the only available electronic states with consistent energy and momenta arise from a conduction band of negative curvature^6,11^.

## Results

### Dipole-allowed transition from high-lying conduction bands

**Fig. 1:**
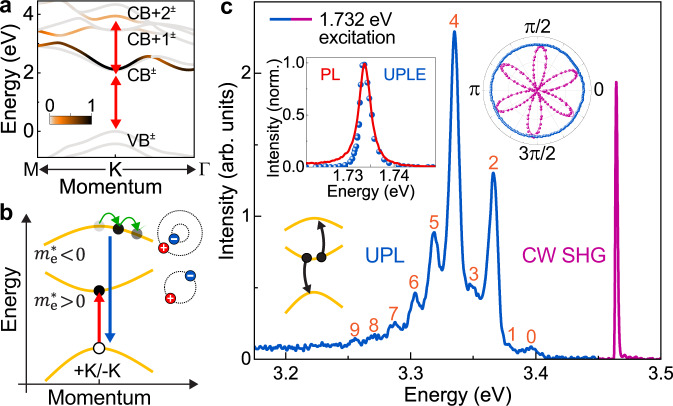
Formation of high-lying excitons (HX) in monolayer WSe_2_, with an electron in an upper conduction band and a hole in the top-most valence band. **a** Calculated *GW* band structure of bare monolayer WSe_2_. Higher (lower) energy spin-split bands are labeled by + (–) superscripts. The color indicates normalized oscillator strength of electrons making a transition from the top valence band (VB^+^) to the different conduction bands (CB, CB+1, and CB+2). Red double-headed arrows mark the exciton resonances invoked to explain quantum interference in optical second-harmonic generation (SHG)^7^. **b** Resonant pumping of band-edge A excitons (red arrow) results in momentum-selective excitation of higher-lying bands at the *K*-symmetry points. Electrons from the high-lying band recombine with holes from the valence band to emit UV light (blue arrow), measured as upconverted photoluminescence (UPL) spectra. Electrons in the first conduction band (CB) relax towards the *K*-points by emitting phonons, whereas electrons in the downwards-curved higher-lying conduction band relax away from the *K*-points (green curves). **c** PL spectrum of hBN-encapsulated monolayer WSe_2_ at 5 K under narrow-band continuous-wave (CW) excitation at 1.732 eV. The peak at 3.464 eV arises from SHG (purple), below which upconverted PL (UPL) from the HX (blue) is observed in the form of a set of 10 peaks. The bottom-left inset indicates the Auger-like process responsible for populating exciton states from the higher-lying conduction band, generating UPL. The intensity of the SHG (purple dots) co-polarized with the laser varies as the laser polarization is rotated with respect to the crystal lattice (right inset), whereas the HX luminescence (blue dots) does not. The top-left inset shows the UPL excitation (UPLE) spectrum of the HX (blue), which matches the band-edge A-exciton PL spectrum (red).

Figure 1a shows the *ab initio GW* quasiparticle band structure of monolayer WSe_2_ around the *K*-point in momentum space. The magnitude of the oscillator strength of the interband transition coupling the spin–orbit–split top valence band to the different conduction bands is coded in color. The full band structure is shown in Supplementary Fig. 1. The lowest-energy band-edge “A-exciton” is characterized by a dipole-allowed optical transition between the valence-band maximum and the upper spin-split conduction-band minimum at the *K*-point in the Brillouin zone. Besides the band-edge transition, our calculations in Fig. 1a indicate an additional dipole-allowed transition at the same *K*-point from a high-lying conduction band (CB+2^−^) to the top valence band (VB^+^)^6,11^, with approximately 4% of the oscillator strength of the band-edge transition (CB^+^ to VB^+^). Such higher-energy transitions happen to be located at around twice the band-edge transition energy (red double-headed arrows).

### Probing the high-lying exciton by momentum-selective UPL

As illustrated in Fig. 1b, to selectively excite electron–hole pairs in the vicinity of the fundamental bandgap at the *K*-points (+*K* or −*K*-point), we use a linearly polarized narrow-band continuous-wave (CW) laser at 716 nm (1.732 eV), resonantly driving the A-exciton of monolayer WSe_2_ encapsulated in hexagonal boron nitride (hBN) (see Supplementary Fig. 3 for sample details). The process of Auger-like exciton–exciton annihilation^8,12–14^, indicated by the sketch in the bottom-left inset of Fig. 1c, can raise the electron to a higher band as momentum conservation localizes it around the *K*-points. The resulting UPL spectrum at 5 K in Fig. 1c shows two distinct features: a narrow peak at twice the excitation energy, i.e. at 3.464 eV; and a series of peaks beginning 60 meV lower in energy. The first peak, limited in width by the spectral resolution of the monochromator, arises from CW SHG^15^, which originates from the broken inversion symmetry of monolayer WSe_2_. The remaining peaks are reminiscent of excitonic luminescence. A total of ten narrow peaks are resolved with a mean linewidth of 11.4 meV. The SHG and excitonic UPL are easily discriminated by measuring the change in emission intensity co-polarized with the laser as the laser polarization is rotated with respect to the WSe_2_ crystal^16^. The right inset of Fig. 1c shows the characteristic six-fold symmetry of the SHG, arising from the three-fold rotational crystal symmetry. In contrast, the polarization dependence of copolarized UPL appears as a circle. Since the UPL arises at energies that are independent of the excitation energy (Supplementary Fig. 4b), the emission cannot be attributed to hyper-Raman scattering and is not related to the phonon cascades recently observed in Raman scattering from monolayer WSe_2_^17^. We label this UV emissive species the “high-lying exciton” HX. The linewidth of the dominant HX peak can be as narrow as 5.8 meV at low pump fluences (see Supplementary Fig. 3d), and therefore places a lower limit on the exciton coherence time of ~100 fs.

### Narrow linewidth and in-plane dipole orientation of the HX

**Fig. 2:**
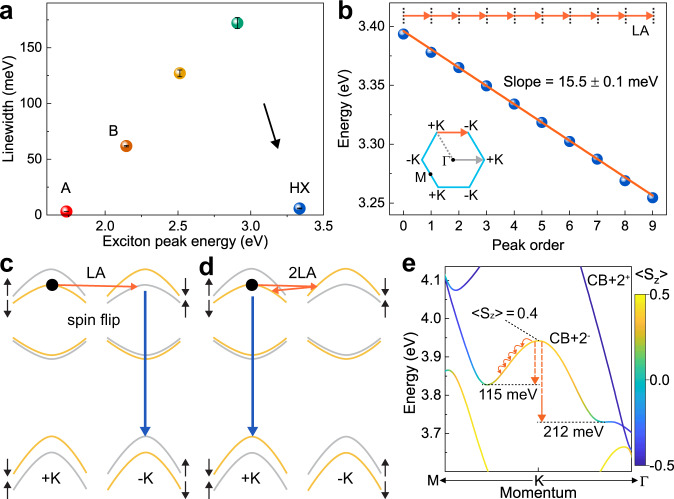
Excitonic linewidths, equidistant phonon progression, and phonon scattering mechanisms in high-lying exciton PL. **a** Energies and linewidths (full width at half maximum) of the prominent excitonic states seen in the PL of hBN encapsulated monolayer WSe_2_. The PL linewidth increases with transition energy until the HX breaks the trend. A summary of the data is given in Supplementary Table 1. The error bars were obtained from peak fitting. **b** HX UPL peak positions as a function of peak number. The error bars were obtained from multi-peak fitting and are below the diameter of the spheres. The fitted slope of 15.5 ± 0.1 meV corresponds to the calculated longitudinal acoustic (LA) phonon energy (orange arrows) near the *K*-points, which allows inelastic scattering of electronic states between +*K* and −*K*-points. Inset: the momentum change from $\Gamma$ to +*K* (gray arrow) equals that from +*K* to −*K* (orange arrow). **c** and **d** The spin (black arrows) sublevels of conduction and valence bands of single-layer WSe_2_ are non-degenerate at the *K*-points. Momentum-conserving electron–phonon scattering (orange arrows) can involve either one phonon and a spin flip (**c**), or a spin-conserving two-phonon double-resonance transition (**d**). **e** Calculated *GW* band structure of monolayer WSe_2_ of CB+2^−^ around the *K*-point. The color code of the band structure shows the expectation values of the spin operator on the spinor wavefunctions in the out-of-plane direction, i.e., 〈*S*_*z*_〉, in units of $\hslash$. The curved arrows illustrate the energy associated with LA-phonon scattering.

Resolving such narrow discrete luminescence peaks at almost twice the bandgap is unexpected since the excitonic linewidth usually increases with excitation or emission energy as shown in Fig. 2a and Supplementary Table 1; higher-lying transitions such as the B and C excitons^18^ are subject to a broader range of non-radiative relaxation channels which limit their lifetime. The relationship between the HX UPL and the A-exciton transition is identified by sweeping the laser energy over the A-exciton resonance while recording the HX luminescence intensity, i.e. performing PL excitation (PLE) spectroscopy as shown in Supplementary Fig. 4b. The top-left inset of Fig. 1c shows the PL spectrum of the fundamental band-edge exciton (red line) in comparison to the PLE spectrum of the integrated HX emission (blue dots); PL and PLE spectra are virtually identical. As we demonstrate in Supplementary Fig. 5, the HX luminescence shows characteristics of in-plane transition-dipole orientation analogous to that of the A-exciton, but unlike the spin-forbidden dark exciton which has an out-of-plane transition dipole^5^.

### Phonon progression in the HX UPL

Figure 2b shows the energy of the HX luminescence peaks as fitted in Supplementary Fig. 6c. The 10 peaks are spaced equally, suggesting that they result from cascaded electron–phonon scattering at a phonon energy of 15.5 ± 0.1 meV. Such a phonon progression is known from PLE spectra^19,20^ and Raman scattering^17,21^ of monolayer TMDCs, but it is very unusual to find it so prominently in their PL spectra, with 10 peaks resolved. Calculations of the phonon dispersion in monolayer WSe_2_ (Supplementary Fig. 1b) suggest that the progression interval corresponds exactly to the emission of a longitudinal acoustic (LA) phonon close to the M and *K*-points of the Brillouin zone, where the phonon density of states is the largest^22^. With increasing temperature, the phonon occupancy (and consequently the electron–phonon interaction) increases and the intensity of the higher-order phonon progression peaks, therefore, increases (Supplementary Fig. 7). We propose that strong inelastic resonant electron–phonon scattering occurs between +*K* and −*K*-valleys^23,24^, as sketched in Fig. 2c, d. Such intervalley scattering can occur in <100 fs^25–27^, while the radiative decay of the band-edge A-exciton in monolayer WSe_2_ takes ~150 fs^4^. Given that the oscillator strength of the HX is expected (and confirmed in our *GW*-BSE calculations) to be much smaller than that of the A-exciton, the radiative decay of the HX is presumably slower.

As in previous studies of monolayer TMDCs, our *GW* band-structure calculations of monolayer WSe_2_ in Supplementary Fig. 1a reveal that all the conduction and valence bands around the *K*-points are spin split because of spin–orbit coupling. The combination of mirror-plane and time-reversal symmetry suggests that spin in the out-of-plane direction, *S*_*z*_, is a good quantum number with opposite orientations in the +*K* and −*K*-valleys^28,29^. Figure 2e shows the CB+2^−^ band, color-coded by the calculated 〈*S*_*z*_〉 expectation value. Two different phonon-scattering processes between valleys are thus conceivable to move an electron of the high-lying exciton downwards in energy in two distinct ways: one-phonon scattering (Fig. 2c), which requires a spin–flip^30,31^; and a double-resonance two-phonon mechanism (Fig. 2d)^23^ conserving spin. The spin-conserving two-photon process should be favored in luminescence over the one-phonon mechanism. Indeed, the spectrum in Fig. 1c demonstrates that even-numbered peaks are more intense than odd-numbered ones, implying a higher transition probability for the two-phonon processes. However, we note that the helicity of the excitation can be destroyed by the fast exchange interaction^32,33^ arising during the Auger-like population process of the HX state so that the luminescence is not necessarily expected to be circularly polarized.

As illustrated in Fig. 1b, repeated electron–phonon scattering in a band of negative curvature should move the electron lower in energy, away from the *K*-symmetric points. Spin-valley locking is then relaxed, which, for higher-lying bands, persists only over a limited region of momentum space close to the *K*-point (see Fig. 2e). The alternation in peak intensity in the HX PL is pronounced up to peak number 5. Inspection of the Brillouin zone of single-layer WSe_2_, inset in Fig. 2b, in conjunction with the phonon progression observed, supports the assertion that the HX PL originates from the *K*-symmetric points. If the radiative state were instead formed around the Γ or *M* points, which lack significant spin-valley locking, there would be no obvious reason for the peak intensity to alternate in the progression. It is conceivable that the HX phonon progression could be interpreted in the context of polaronic excitons, which may provide an explanation for the extraordinarily narrow linewidth of the luminescence. The question of whether the progression is best rationalized in terms of bound excitons which emit phonons, or as polaronic excitons which form into split subbands^24^, is primarily a matter of the electron–phonon coupling strength, offering challenges for future study.

### *p*-like HX state in two-photon PL excitation spectroscopy

**Fig. 3:**
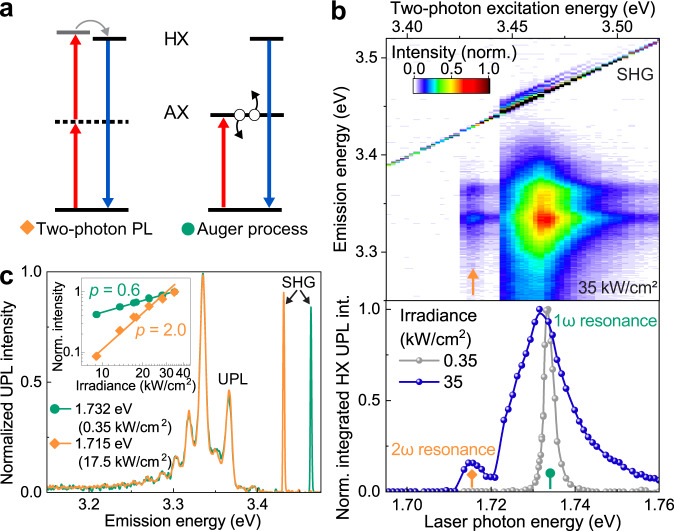
Excitation pathways of the high-lying exciton HX. **a** The HX can be formed either by two-photon absorption (TPA) involving a virtual level (dashed) or by Auger-like exciton-exciton annihilation (black arrows) of the A-exciton AX. TPA populates a dark *p*-like HX state, which subsequently relaxes (gray arrow) to the bright *s*-like HX state. **b** Change of the UPL spectrum with a photon energy of the CW laser at an irradiance of 35 kW/cm^2^, showing TPA (arrow) below the onset of the Auger-like double-exciton process. The diagonal line stems from SHG from the WSe_2_ monolayer. The spectrally integrated HX UPL intensity is plotted as a function of excitation energy in the lower panel. TPA (2${{{{{\rm{\omega }}}}}}$ resonance) is observed at a pump intensity of 35 kW/cm^2^, while only the one-photon (1${{{{{\rm{\omega }}}}}}$) resonance is observed at 0.35 kW/cm^2^. **c** The two excitation mechanisms are distinguished by their power dependencies (inset). For the Auger-like mechanism (green), excitation in resonance with the band-edge A-exciton at 1.732 eV gives a sublinear dependence of UPL intensity on excitation power because of bleaching of the A-exciton transition. The corresponding power-law exponent *p* is about 0.6. Sub-resonant excitation at 1.715 eV yields the parabolic power dependence characteristic of TPA (orange). The corresponding power-law exponent is close to 2.0. The HX PL spectrum is independent of the excitation pathway (green and orange lines).

A signature of the excitonic nature of optical transitions in TMDC monolayers is the existence of a series of excited states with different selection rules^5,34^. Besides Auger-like excitation, two-photon absorption (TPA) may also populate a dark excitonic state that subsequently converts to the radiative HX state. As sketched in Fig. 3a, for systems undergoing dipole-allowed interband transitions with winding number zero^34^, TPA probes odd-parity “*p*-like” excitonic states, while one-photon absorption and emission correspond to transitions involving even-parity “*s*-like” states^34–36^. For the excitation formed by TPA to relax to the radiative HX, the TPA energy must be no lower than the HX zero-phonon transition energy, i.e. around 3.4 eV. There is no need for TPA to occur at exactly twice the energy of the A-exciton, i.e. to overlap with the one-photon resonant Auger-like mechanism identified in the PLE in Fig. 1c. Figure 3b plots emission spectra as a function of excitation energy for a high pump fluence of 35 kW/cm^2^, revealing a clear cut-off just below the transition energy of the A-exciton. TPA (orange arrow) sets in when the laser energy is just below the A-exciton transition (green marker). The diagonal line stems from the SHG. Auger and TPA mechanisms are clearly distinguished by their respective power dependencies shown in the inset of Fig. 3c: the intensity of two-photon PL (orange) scales quadratically with excitation power, whereas the Auger-like process (green) shows a sublinear dependence because of bleaching of the A-exciton transition at high powers^8^. Although the two PLE features are not degenerate, the PL spectra from TPA and Auger-like excitation are identical (panel c). The lower part of Fig. 3b illustrates the pump-power dependence of the PLE spectra, obtained from the integrated HX PL intensity as a function of excitation energy. At low powers, only one PLE peak is seen, corresponding to the PL spectrum of the A-exciton and implying Auger-assisted upconversion to the HX as described in Fig. 1c. At high powers, this feature broadens, presumably because of electron–electron scattering and increased screening of the excitonic electron–hole pair, and an additional peak appears due to TPA. This narrow PLE peak has a linewidth of ~12 meV, virtually identical to the average linewidth of the HX PL transitions in Fig. 3c, thus suggesting that TPA and PL transitions arise from excitonic states of similar nature. The complete evolution of the PLE spectrum with pump power is shown in Supplementary Fig. 4. As we discuss in Supplementary Fig. 2, the energy of the non-emissive *p*-like HX state probed by TPA (3.43 eV) matches perfectly with the energy at which the transparency dip is observed in SHG (3.423 eV) due to the excitonic quantum interference effect^7^. This coincidence also implies that the HX cannot arise from a localized defect in the WSe_2_ monolayer crystal^37,38^.

### Sample to sample variation of HX energy

To prove that the two-photon excitation and the one-photon emission of the HX do not simply originate from different phonon transitions of one and the same state, we also performed measurements on monolayer WSe_2_ encapsulated by hBN layers of different thicknesses. Changing the dielectric environment of the monolayer modifies the binding energy of excitons and thus the energy separation between excitonic states^5^, but should not affect intralayer phonons. As demonstrated in Supplementary Fig. 6 and Table 1, the separation between *p*-like HX states, probed by TPA, and *s*-like HX states, probed by HX PL, varies between 27 and 37 meV across samples. No such variation is seen in the spacing of the phonon progression, which is identical for all samples (Supplementary Table 2).

### Ab initio *GW*-BSE calculation of the HX

**Fig. 4:**
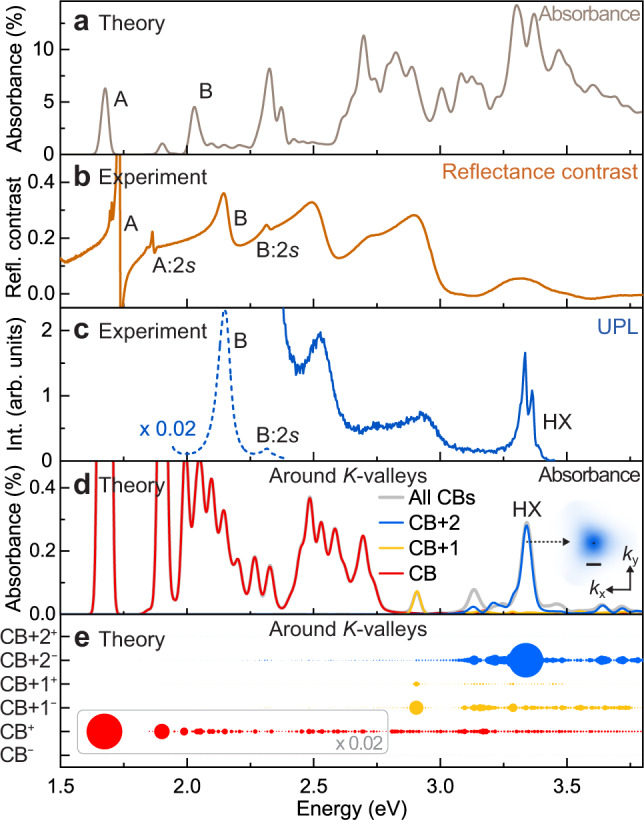
Identification of the bound HX in ab initio *GW*-BSE calculations. **a** Calculated absorbance spectrum of bare monolayer WSe_2_, accounting for transitions over the entire Brillouin zone. The dominant low-energy excitonic transitions are indicated. **b** Experimental reflectance contrast of monolayer WSe_2_ encapsulated by hBN on a sapphire substrate at 5 K. **c** UPL spectrum (solid line) measured under resonant pumping of the A-exciton. The dashed line shows the same spectrum multiplied by 0.02 at the corresponding energy range. **d** The calculated absorbance spectrum of bare monolayer WSe_2_, restricted to transitions around the *K*-points (within 6% of the Brillouin-zone area) from the topmost VB^+^ to only the lower conduction bands (CB, red), only the CB+1 bands (yellow), only the CB+2 bands (blue), or all bands (gray). The HX feature at 3.34 eV appears only when contributions from CB+2 are included. The inset shows the square of the HX envelope function in reciprocal space centered around K (black dot), with a scale bar of 0.2 ${\rm A} ^{-1}$. **e** Contributions of the different spin-split conduction bands to the excitonic optical strength of the different peaks in (**d**), indicated by the size of the disk area. The dominant contribution to HX comes from transitions from the VB^+^ to the negative-mass band CB+2^−^. The disk area within the gray box is rescaled by a factor of 0.02.

To conclusively establish the contribution of higher-lying conduction bands to the HX, we performed ab initio *GW*-BSE calculations^18,39,40^, as implemented in the BerkeleyGW package^41^. Figure 4a shows the computed absorbance spectrum of monolayer WSe_2_. The calculation explains the salient features (which are shown theoretically to be excitonic transitions in the absorbance) of the experimental reflectance contrast of hBN-encapsulated WSe_2_ plotted in Fig. 4b. The minor difference between the calculated and measured energies of excitonic transitions stems mainly from the influence of the dielectric environment, i.e. the hBN encapsulation, which is not included in the calculation. We discuss the influence of the hBN layers on the calculated exciton transition energy in Supplementary Fig. 8. As seen from Fig. 4a, b, there are many features around 3.4 eV, i.e. in the range of the HX PL and TPA. These features contain different transitions involving different bands and *K*-points throughout the Brillouin zone. Given that the interband transition from a high-lying conduction band (CB+2^−^) to the top valence band (VB^+^) at the *K*-point has only ~4% of the oscillator strength of the interband transition of the band-edge A-exciton transition (CB^+^ to VB^+^), the narrow-linewidth HX is not expected to be discernible in conventional absorption or reflectance measurements. We emphasize that this multitude of transitions arises because both the calculated absorbance and the experimental reflectance probe the entire Brillouin zone and therefore sample different bands. Resonant pumping of A-excitons in UPL, however, selectively populates excitons, including the HX, around the *K*-valleys. Figure 4c plots the entire UPL spectrum, showing the narrow HX feature together with several broader excitonic features at lower energies. To identify the HX in the calculation, we filter out excitons originating from transitions outside the *K*-valleys, retaining only those formed by transitions within a range of <0.2 Å^−1^ around the *K*-point as illustrated in Supplementary Fig. 1a. Since pumping A-excitons predominantly populates holes at the topmost valence band, we only include this band (VB^+^) in the calculation here for clarity. Excitons with contributions from both VB^+^ and VB^−^ are shown in Supplementary Fig. 9. The resulting absorbance spectrum, restricted to transitions around the *K*-valleys, is plotted in Fig. 4d. The HX peak at 3.34 eV clearly stands out in the calculation at the UV range, with no comparable peaks apparent within the ±0.5 eV energy range.

We also show calculated absorbance spectra when the transitions include only a subset of the conduction bands: only CB (red), only CB+1 (yellow), or only CB+2 (blue). The calculation restricted to only CB+2 closely reproduces the HX feature obtained when all CBs are included. The contributions of individual conduction bands to the wavefunction of the excitons with different energy in the *K*-valleys are examined further shown in Fig. 4e, where the area of each disk plotted is proportional to the integrated oscillator strength of the different band components (see Supplementary Methods and Notes for details). From these analyses, it is evident that the optical strength of the high-lying feature HX arises primarily from the dominant contribution of the downcurved conduction band CB+2^−^, offering a clear rationalization of the experimental observations in Fig. 1. As we discuss in detail in Supplementary Fig. 10, the HX also contains an admixture of the CB+1 bands. This constituent is non-emissive (hence does not show up clearly in Fig. 4e) and may, therefore, contribute to the narrow linewidth exhibited by the HX PL. The lower conduction bands have a much smaller contribution to the HX peak due to the relatively large energy separation between higher (CB+2) and lower conduction bands (CB and CB+1) in the *K*-valley.

### Excitons comprising negative-mass electrons

The band structure in Fig. 1a and Supplementary Fig. 1a shows that CB+2^−^ corresponds to an electron of negative mass. The inset in Fig. 4d shows the envelope function of the HX wavefunction in momentum space, which is to be compared with the A-exciton wavefunction in Supplementary Fig. 10. In Supplementary Fig. 10e we project the HX exciton envelope function onto the quasiparticle band structure to demonstrate that the HX is indeed localized around the *K*-points, where the effective-mass approximation remains valid. An effective two-band model as shown in Supplementary Fig. 11 gives the same conclusion that the HX really can reside around the *K*-points in momentum space instead of spreading out to local minima of the high-lying conduction band. The HX has a calculated exciton radius^5^ of 1.2 nm, which is smaller than the 1.5-nm radius of the 1*s* A-exciton. This reduction is consistent with a heavier reduced exciton mass arising from an electron in a band of negative curvature, i.e. of negative effective mass.

## Discussion

The idea of a stable exciton involving a negative-mass electron may seem counterintuitive. However, such a complex is indeed possible within a simple effective-mass hydrogenic model, provided that the hole mass ${m}_{{{{{{\rm{h}}}}}}}^{\ast }$ is *positive* and smaller in magnitude than the negative electron mass ${m}_{{{{{{\rm{e}}}}}}}^{\ast }$ (see Supplementary Fig. 12) so that the reduced mass of the exciton ($1/\mu =1/{m}_{{{{{{\rm{h}}}}}}}^{\ast }+1/{m}_{{{{{{\rm{e}}}}}}}^{\ast }$) remains positive. Then, the total mass of the exciton ($M={m}_{{{{{{\rm{h}}}}}}}^{\ast }+{m}_{{{{{{\rm{e}}}}}}}^{\ast }$) becomes negative. Drawing an analogy to classical orbital motion, the semiclassical motion of the electron and hole may then be thought of as joint orbiting around a common center which does not lie geometrically between the two particles, so that, as indicated on the top right side of Fig. 1b, the electron accelerates in the same direction as the hole. It is important to stress that, because the HX forms around the *K*-point, such an exciton is not related to the so-called “camel’s back exciton” formed due to a local camel-back-like structure in the conduction band^42^, or the somewhat more controversial so-called “bielectron” state^43–45^. In this proposed bound bielectron model, a negative-mass electron and a positive-mass electron from different conduction bands are bound together under the condition of the negative-mass electron being lighter.

The observation of an alternating peak intensity in the HX phonon progression (Fig. 1c) is consistent with the involvement of a negative-mass electron undergoing a cascaded phonon emission away from the *K*-points as sketched in Fig. 1b. If, instead, the HX were formed by positive-mass electrons, phonon emission would move electrons towards the *K*-points, where the spin-valley locking is more pronounced. The alternating behavior in the intensity of the phonon peaks would then appear stronger with increasing peak order, in conflict with the observation in Fig. 1c. Direct experimental evidence for the negative total mass of this peculiar excitonic species remains subject to further research. Studies of their ultrafast dynamics, diffusion and magneto-optical characteristics promise to be particularly insightful in this regard.

Since the electronic structure of many semiconducting monolayer TMDCs is qualitatively comparable^6,11^ and the efficient upconversion process has been observed in both monolayer MoSe_2_ and MoTe_2_^14^, narrowband high-lying excitons in the free-particle continuum are expected to be a generic feature of TMDCs. Our results unveil the rich physics of complex many-body excitations in monolayer TMDCs, which emerges not only in the formation of various excitons and exciton complexes but, more importantly, in the coherent coupling and interference between excitonic transitions. In comparison to band-edge excitons, high-lying excitons appear to have larger orbital contributions from the chalcogenide atoms and are therefore much more sensitive to band hybridization with an adjacent layer in multilayer structures^46^. As we recently proved in twisted-bilayer WSe_2_, the HX energy can be tuned over a broad range by twist angle, with an average twist-angle susceptibility of 8.1 meV/°. Correspondingly, the excitonic quantum interference can be turned on and off by twisting^46^. High-lying excitons combined with band-edge excitons, therefore, form an atomic-like excitonic multilevel system, setting the basis for future exploration of quantum excitonics.

## Methods

### Sample preparation

We exfoliated the monolayer WSe_2_ and thin layers of hBN from bulk crystals (WSe_2_, HQ Graphene; hBN, NIMS) on PDMS films (Gel-Pak, Gel-film^®^ X4) using Nitto tape (Nitto Denko, SPV 224P)^47^. We stamp-transferred the hBN and monolayer WSe_2_ flakes layer by layer onto either Si/SiO_2_, sapphire, or diamond substrates while heating the substrate to 65 °C. The hBN-encapsulated WSe_2_ samples were annealed under a high vacuum at 150 °C for 5 h.

### Optical spectroscopy

The setup is illustrated in Supplementary Fig. 13a. We used an objective of 0.6 numerical aperture (Olympus, LUCPLFLN) to focus the laser onto the sample and collect the signal. The sample was placed under a vacuum on the cold finger of a helium-flow cryostat (Janis, ST-500). We used a grating of 1200, 600 or 150 grooves mm^−1^ to disperse the signals, and a CCD camera (Princeton Instruments, PIXIS 100) to record the spectra. A 50:50 beam splitter was used to separate excitation and detection pathways. The photon-energy-dependent instrument response in Supplementary Fig. 13b was measured with a calibration light source (LS-1-CAL, Ocean Optics) placed in front of the objective. The original spectra are shown in the main text and the Supplementary Information, without correction for the instrument response. We measured the photoluminescence (PL) of monolayer WSe_2_ by exciting samples with an argon-ion laser (Spectra Physics, 2045E) at 488 nm and filtering out the laser line from the signal with a 488 nm long-pass edge filter. The reflectance contrast of monolayer WSe_2_ was measured using a broad-band Xenon lamp (EQ-99X, Energetiq). We measured the upconverted PL and SHG of monolayer WSe_2_ by exciting samples with a tunable CW laser (Sirah, Matisse CR) and filtering out the laser line using a 680 nm short-pass filter.

## Supplementary information

Supplementary Information

Peer Review File

## Data Availability

The raw data that support the plots within this paper and the other findings of this study are available from the corresponding authors upon reasonable request.
