# Supplementary material for: Narrow-band high-lying excitons with negative-mass electrons in monolayer WSe2
Source: Nat Commun. 2021 Sep 17;12:5500. doi: 10.1038/s41467-021-25499-2 (PMC8448890; doi:10.1038/s41467-021-25499-2)
Supplement: Supplementary file 1 — Supplementary Information [file 41467_2021_25499_MOESM1_ESM.pdf]

Narrow-band high-lying excitons with negative-mass electrons in monolayer WSe_2

- Supplementary Information -

Kai-Qiang Lin, Chin Shen Ong, Sebastian Bange, Paulo E. Faria Junior, Bo Peng, Jonas D. Ziegler, Jonas Zipfel, Christian Bäuml, Nicola Paradiso, Kenji Watanabe, Takashi Taniguchi, Christoph Strunk, Bartomeu Monserrat, Jaroslav Fabian, Alexey Chernikov, Diana Y. Qiu, Steven G. Louie, John M. Lupton

Correspondence to: kaiqiang.lin@ur.de, john.lupton@ur.de

Contents:

Supplementary Methods and Notes
Supplementary Figures 1-13
Supplementary Tables 1-2
Supplementary References

Supplementary Methods and Notes

<u>*Ab initio GW* and *GW*-BSE Calculations</u>

Density-functional theory (DFT) Kohn-Sham wavefunctions for monolayer WSe_2 are calculated using the Quantum ESPRESSO[1] package as the starting orbital energies and wavefunctions for our *GW* calculation. For the DFT calculations, we use a plane-wave basis set and norm-conserving pseudopotentials. Scalar-relativistic (SR) pseudopotentials are used for spin-unpolarized scalar-relativistic calculations, and fully relativistic (FR) pseudopotentials are used for fully relativistic noncollinear spinor calculations. The generalized gradient approximation (GGA-PBE)[2-4] is used for the electron exchange and correlation energy. To accurately capture the exchange contribution to the *GW* quasiparticle (QP) self-energy of the monolayer WSe_2, semi-core 5*s*, 5*p*, and 5*d* states are included in the pseudopotentials of W in addition to the 6*s* and 6*p* valence electrons. The plane-wave cutoff for the DFT calculation is set to 80 Ry for the plane-wave expansion of the wavefunctions. The length of the periodic supercell is set to be L_z = 120 Å. The crystal structure of monolayer WSe_2[5] has in-plane lattice constants of 3.2820 Å and an atomic-plane to atomic-plane Se-Se distance of 3.3411 Å.

The quasiparticle (QP) self-energies and exciton excitation energies are, respectively, computed using the *ab initio GW* and *GW* plus Bethe-Salpeter equation (*GW*-BSE) approaches as implemented in the BerkeleyGW[6-8] package. In the *GW* and *GW*-BSE calculations, the Coulomb interaction beyond 60 Å in the *z*-direction (i.e., out-of-plane direction) is truncated to prevent spurious interactions between periodic images[9]. The calculated QP band structure is plotted in Fig. 1a and Supplementary Fig. 1.

To calculate the excitation energies of the excitons, the Bethe-Salpeter equation (BSE)[7,8] is solved by using the above-calculated QP energies and spinor DFT wavefunctions. The electron-hole interaction kernel of the BSE Hamiltonian is first calculated on a uniform **k**-grid of 72×72, using two valence and eight conduction bands and a dielectric matrix that is calculated on a uniform **q**-grid of 72×72, summed over 1400 bands and using a 5-Ry-plane-wave cutoff. The BSE Hamiltonian is diagonalized using interaction kernel matrix elements that are interpolated[7,10] from the uniform 72×72 k-grid to a finer uniform k-grid of 120×120, using directly calculated matrix elements for **q**-points of a density equivalent to 150×150. Using the calculated exciton wavefunctions and excitation energies, the imaginary part of the dielectric function is calculated using $\mathrm{Im}[\epsilon(\omega)] = \frac{8\pi^2 e^2}{\omega^2}\frac{1}{V}\sum_S |\mathbf{e}\cdot\langle 0|\mathbf{v}|S\rangle|^2 \delta(\omega-\Omega^S)$, where $\langle 0|\mathbf{v}|S\rangle$ is the optical velocity matrix element between exciton $|S\rangle$ and ground state $|0\rangle$, Ω^S is the energy of state $|S\rangle$, $\mathbf{e}$ is the direction of the polarization of light, and V is the crystal volume. The velocity matrix element of the exciton, $\langle 0|\mathbf{v}|S\rangle$, is a coherent superposition of transitions between non-interacting electron-hole pairs $\langle v\mathbf{k}|\mathbf{v}|c\mathbf{k}\rangle$, given by $\langle 0|\mathbf{v}|S\rangle = -\Omega^S \sum_{vc\mathbf{k}}^{S}(A_{vc\mathbf{k}}^{S}\frac{\langle v\mathbf{k}|\mathbf{v}|c\mathbf{k}\rangle}{E_{v\mathbf{k}}-E_{c\mathbf{k}}})$, where $E_{n\mathbf{k}}$ and $|n\mathbf{k}\rangle$ are the Kohn-Sham eigenvalues and eigenfunctions, respectively. To obtain the absorption spectrum from a selectively excited part of momentum space, we calculate the mode-decomposed (MD) imaginary part of the dielectric function, $\mathrm{Im}[\epsilon(\omega)]_{\mathrm{MD}} = \frac{8\pi^2 e^2}{\omega^2}\frac{1}{V}\sum_S |\mathbf{e}\cdot\langle 0|\mathbf{v}|S\rangle_{\mathrm{MD}}|^2 \delta(\omega-\Omega^S)$, where $\langle 0|\mathbf{v}|S\rangle_{\mathrm{MD}} = -\Omega^S \sum_{vc\mathbf{k}\in\mathrm{subset}}^{S}(A_{vc\mathbf{k}}^{S}\frac{\langle v\mathbf{k}|\mathbf{v}|c\mathbf{k}\rangle}{E_{v\mathbf{k}}-E_{c\mathbf{k}}})$, i.e., the band- and k-resolved contributions to the dielectric function. To calculate the absorbance, the real part of the dielectric function is firstly obtained from the imaginary part using the Kramers-Kronig relation. Using the dielectric function $\epsilon(\omega)$, we calculate the extinction coefficient $\kappa(\omega)$. Finally, the absorbance spectra $\mathrm{A}(\omega)$,

as defined by $A(\omega) = \log_{10}[I(\omega)/I_0(\omega)] \times 100\%$, are plotted in Fig. 4 of the main text and Supplementary Fig. 9.

In Fig. 4d, the plots are made for contributions to the absorbance due to the subset of $vc\mathbf{k}$ that corresponds to parts of momentum space that are circular patches of 0.2 $Å^{-1}$ radius centered at the K-valleys (highlighted in Supplementary Fig. 1a), involving only transitions from the highest valence band. In Fig. 4e, we plot the mode-decomposed oscillator strengths for each contributing conduction band. The area of each disk in Fig. 4e is proportional to the integrated oscillator strengths from the relevant modes of the contributing excitons, where the oscillator strength of each excitonic state, $|S\rangle$, is given by $f_S = \frac{2|\mathbf{e}\cdot\langle 0|\mathbf{v}|S\rangle_{\mathrm{MD}}|^2}{\Omega^S}$. We integrate only the subset of $vc\mathbf{k}$ that contributes to the oscillator strength. To analyze the exciton radius, we calculate the root mean square radius of the exciton envelope function in real space. In Supplementary Fig. 10c, we show the momentum-space envelope functions of three individual excitons arising primarily from CB, CB+1, and CB+2 in the vicinity of the K-valleys. Notably, the CB band curvature is smaller than the CB+1 band curvature, and CB+2 has negative curvature. Therefore, the reduced mass of the CB exciton is smaller than that of the CB+1 exciton, and the reduced mass of the CB+1 exciton is smaller than that of the CB+2 exciton (HX). This increase in mass is consistent with the fact that the CB exciton has a larger exciton radius than the CB+1 exciton and the CB+1 exciton has a larger exciton radius than the CB+2 exciton. The CB+2 exciton also has a larger binding energy of 0.6 eV than the CB+1 exciton (0.55 eV) and the CB exciton (0.45 eV). These values will decrease with hBN encapsulation because of increased dielectric screening.

Effective-mass model

In order to investigate the stability of the HX within the effective-mass limit, we restrict ourselves to the two bands with the largest contribution, i.e. VB^+ and $CB{+}2^-$ around the K-point, based on the *GW*-BSE calculations given in Fig. 1a, Fig. 4 and Supplementary Fig. 1. The effective masses are calculated from the *GW* band structure, leading to $m_\mathrm{h}^* = 0.3636$ for VB^+ and $m_\mathrm{e}^* = -0.4604$ for $CB{+}2^-$. These values fulfill the condition $|m_\mathrm{h}^*| < |m_\mathrm{e}^*|$ and thus the reduced mass of the exciton, $\mu = m_\mathrm{e}^* m_\mathrm{h}^* / (m_\mathrm{e}^* + m_\mathrm{h}^*)$, remains positive. For illustration purposes, we investigate the effect of $|m_\mathrm{h}^*| > |m_\mathrm{e}^*|$ by simply arbitrarily interchanging the two values. The excitonic states are obtained by solving the effective BSE[7,11,12] (*28*, *39-40*), given by

$$[E_\mathrm{e}(\vec{k}) - E_\mathrm{h}(\vec{k}) - \Omega_N]A_N(\vec{k}) + \sum_{\vec{k'}} V(\vec{k} - \vec{k'})\, A_N(\vec{k'}) = 0 \qquad (1),$$

where $E_\mathrm{e}(\vec{k}) = E_0 + \hbar^2 k^2/2m_e^*$, $E_\mathrm{h}(\vec{k}) = -\hbar^2 k^2/2m_\mathrm{h}^*$, and $A_N(\vec{k})$ is the envelope function of the N-th exciton state. The electron-hole interaction $V(\vec{k} - \vec{k'})$ is described by the Rytova-Keldysh potential[13-15]

$$V\left(\vec{k} - \vec{k'}\right) = \frac{-1}{\mathcal{A}} \frac{\mathrm{e}^2}{2\varepsilon_0} \frac{1}{\varepsilon\left|\vec{k} - \vec{k'}\right| + \mathrm{r}_0\left|\vec{k} - \vec{k'}\right|^2} \qquad (2),$$

in which $\mathcal{A}$ is the unit area, e is the electron charge, ε_0 is the vacuum permittivity, r_0 is the screening length of the 2D material, and ε is the effective dielectric constant. For the calculations, we assume a bare WSe_2 monolayer, i.e. $\varepsilon = 1$ and $\mathrm{r}_0 = 45.1$ Å (*44*). We solve the effective BSE numerically in a 2D *k*-grid from -0.5 to 0.5 $Å^{-1}$ in k_x and k_y directions with 121×121 points (leading

to a spacing between k-points of $\Delta k = 0.5/60 \approx 0.0083$ Å^{-1}). To improve convergence, we average the Coulomb potential around each k-point in a square region of $-\Delta k/2$ to $\Delta k/2$ sampled with 121×121 points.

As shown in Supplementary Fig. 11, the effective two-band model gives the same conclusion that the HX can reside around the K-point in momentum space instead of spreading out to local minima of the high-lying conduction band.

In Supplementary Fig. 12a, we present the electronic band structure within the effective-mass approximation for the two conditions $|m_{\mathrm{h}}^*| < |m_{\mathrm{e}}^*|$ (solid blue curves) and $|m_{\mathrm{h}}^*| > |m_{\mathrm{e}}^*|$ (dashed grey curves). In Supplementary Fig. 12b we show the energy difference between conduction and valence bands, $E_{\mathrm{e}}(\vec{k}) - E_{\mathrm{h}}(\vec{k})$, i.e. the diagonal contribution of the BSE equation in Eq. (1). The two different mass conditions thus lead to distinct effective curvatures. From this analysis we can already envision localized excitons around $k = 0$ for $|m_{\mathrm{h}}^*| < |m_{\mathrm{e}}^*|$, whereas for $|m_{\mathrm{h}}^*| > |m_{\mathrm{e}}^*|$ the excitons would be delocalized at the edges of the k-region we considered in the calculations. The fundamental exciton (N = 1) envelope function is shown in Supplementary Fig. 12c, supporting our expectations of bound excitons around $k = 0$ for the condition of $|m_{\mathrm{h}}^*| < |m_{\mathrm{e}}^*|$ and no bound exciton for the condition of $|m_{\mathrm{h}}^*| > |m_{\mathrm{e}}^*|$. We note that the s-like shape of the exciton envelope function obtained within this effective description is consistent with the results obtained from the *ab initio* *GW*-BSE calculations. In order to provide a more realistic comparison between the calculated exciton energies and the experimental data, we consider the influence of hBN encapsulation on the exciton energy in Supplementary Fig. 8 below. This effect can be investigated simply within the effective-mass model by varying the value of the effective dielectric constant ε in the Rytova-Keldysh potential (Eq. 2). The other parameters remain fixed for these calculations.

<u>Phonon calculation and electron-phonon coupling</u>

Based on state-of-the-art DFT, we perform first-principles calculations of the phonons using the Vienna *ab-initio* simulation package (VASP)[16]. We use the projector-augmented-wave potential method with tungsten $5p^6 5d^4 6s^2$ and selenium $4s^2 4p^4$ valence states, together with the PBE-GGA parameterization for the exchange correlation functional[2]. A plane-wave basis set with a kinetic energy cutoff of 500 eV and a 15×15 $\boldsymbol{k}$ mesh over the electronic Brillouin zone (BZ) leads to converged results. For structural relaxation of the unit cell, we consider energy differences converged to within 10^{-6} eV and Hellmann-Feynman forces converged to within 10^{-4} eV/Å. We obtain the harmonic interatomic force constants with density functional perturbation theory using a 5×5 supercell with a $\boldsymbol{k}$-point sampling of 3×3 of the electronic BZ. The phonon dispersion and eigenvectors are calculated using the PHONOPY package[17].

We estimate the strength of electron-phonon coupling by building a smallest-possible non-diagonal supercell that includes the K-point in the vibrational BZ (*47*):

$$\begin{bmatrix} \mathbf{a}_{\mathrm{s}} \\ \mathbf{b}_{\mathrm{s}} \end{bmatrix} = \begin{bmatrix} 1 & 2 \\ 0 & 3 \end{bmatrix} \begin{bmatrix} \mathbf{a}_{\mathrm{p}} \\ \mathbf{b}_{\mathrm{p}} \end{bmatrix} \quad (3),$$

where $\mathbf{a}_{\mathrm{s}}$ and $\mathbf{b}_{\mathrm{s}}$ are the lattice parameters of the supercell, and $\mathbf{a}_{\mathrm{p}}$ and $\mathbf{b}_{\mathrm{p}}$ are the lattice parameters of the unit cell. Using this supercell, we calculate the electronic energy levels at the electronic K-points as a function of the normal mode amplitude u_{qv} of the LA phonon mode at the vibrational K-point. The normal-mode amplitude is defined as[18]

$$u_{\boldsymbol{q}\upsilon} = \frac{1}{\sqrt{N_p}} \sum_{\boldsymbol{R}_p,\alpha,i} \sqrt{m_\alpha} h_{p\alpha i} e^{-i\boldsymbol{q}\cdot\boldsymbol{R}_p} w_{-\boldsymbol{q}\upsilon;i\alpha} \quad (4),$$

where $\boldsymbol{q}$ and υ are reciprocal-space phonon wavevector and branch, respectively, N_p is the number of primitive cells in the real-space supercell, $\boldsymbol{R}_p$ is the position vector of unit cell p, m_α is the nuclear mass of atom α, i runs over Cartesian coordinates, $h_{p\alpha i}$ is the displacement coordinate, and $w_{-\boldsymbol{q}\upsilon;i\alpha}$ is the corresponding eigenvector.

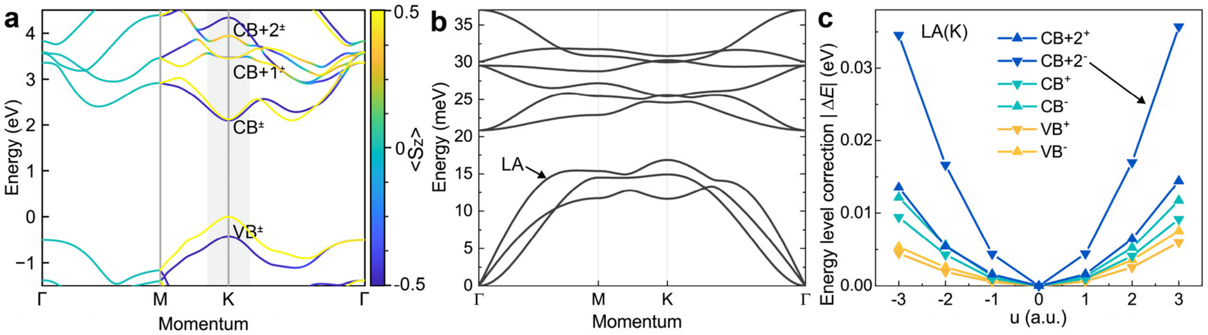

Supplementary Figure 1 | Calculated *GW* band structure, phonon dispersion, and electron-phonon coupling of free-standing monolayer WSe_2. a, *GW* band structure. The bands around the K-point are labelled with CB+2$^+$, CB+2$^-$, CB+1$^+$, CB+1$^-$, CB$^+$, CB$^-$, VB$^+$ and VB$^-$ according to their energy. The grey shading highlights the momentum space used to identify the high-lying exciton (HX) in the absorbance spectrum in Fig. 4d using *GW*-BSE calculations. The color code of the band structure shows the expectation values of the spin operator on the spinor wave-functions in the out-of-plane direction, i.e., $<S_z>$, in units of $\hbar$. Spin-valley locking, as seen in the preservation of the line color, arises in the CB+2 bands, but only over a small region around the K points, substantially smaller than for the lower-energy CB and VB bands. **b**, calculated phonon dispersion. **c**, Energy level correction of electronic states at the K-point with respect to the LA(K) phonon as a function of the amplitude of a specific "frozen" phonon, showing much stronger electron-phonon coupling for electronic states in CB+2$^-$ than in CB$^+$ and VB$^+$. "u" is the normal mode amplitude of the LA(K) phonon as defined in Eq. (4).

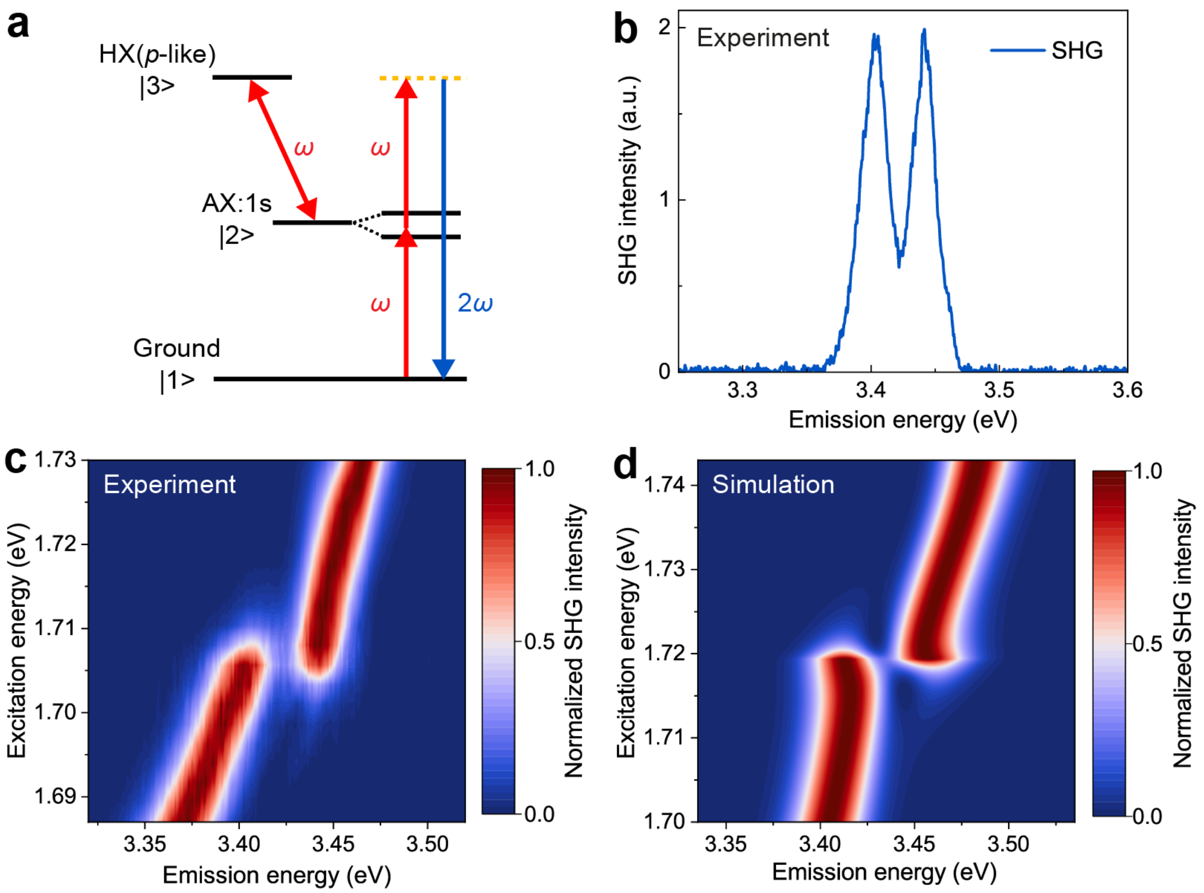

Supplementary Figure 2 | Quantum interference in second-harmonic generation (SHG) from hBN encapsulated WSe_2 monolayer (Sample 0), following Ref. [19]. a, A control beam couples state $|2\rangle$ (A:1*s*) and state $|3\rangle$ (the *p*-like HX), dressing the state $|2\rangle$. The probe beam probes the dressed state via optical SHG. The control and probe beams are degenerate in this case, which resembles degenerated electromagnetically induced transparency (EIT). **b**, SHG spectrum with an transparency dip at 3.423 eV. **c**, Dependence of normalized SHG intensity on excitation energy, measured with an 80-fs pulsed laser. **d**, Simulated normalized SHG intensity as a function of excitation energy with a ladder-type three-level system using the density matrix formalism[19]. The state $|2\rangle$ is set to 1.734 eV according to the photoluminescence (PL) of A:1*s* in Supplementary Fig. 3b. The state $|3\rangle$ is set to 3.43 eV, according to the energy of the *p*-like HX in the PLE measurement in Fig. 4 of the main text. The coherence times of states $|2\rangle$ and $|3\rangle$ are set to match the 3 meV linewidth of the A exciton. In the simulation, the transparency dip in SHG arises close to 3.434 eV, which is about 11 meV above the experimental result. This difference is attributed to bandgap renormalization arising from the strong femtosecond-pulse pumping which is not accounted for in the model.

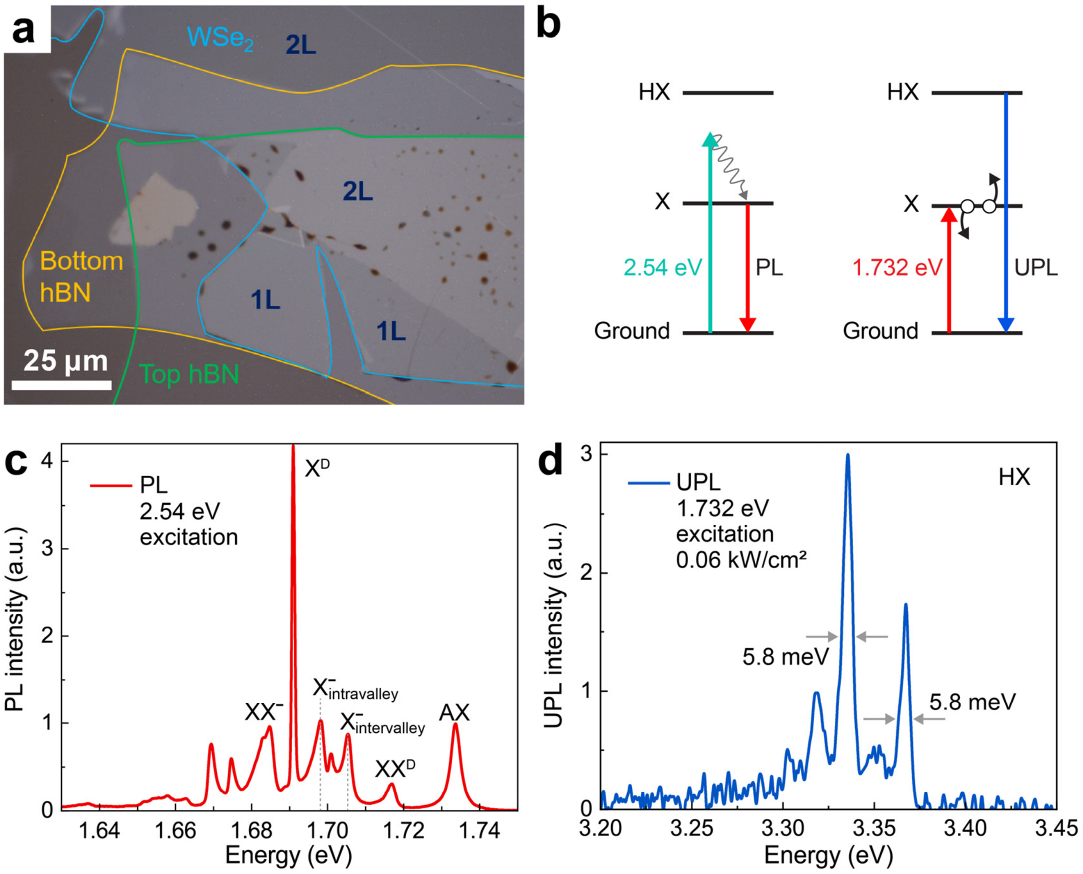

Supplementary Figure 3 | Optical image, PL, and upconverted PL (UPL) of hBN-encapsulated WSe_2 monolayer (Sample 0) presented in the main text. a, Optical microscopic image of the hBN-encapsulated WSe_2 on a sapphire substrate. **b**, Schematic illustration of the PL measurement of the X with laser excitation at 2.54 eV and the UPL measurement of the HX with excitation at 1.732 eV. **c**, PL spectrum of the hBN-encapsulated monolayer WSe_2 in (A) excited by a continuous wave laser at 488 nm. The A-exciton (AX), biexciton (XX^D), trions ($X^-_{intravalley}$, $X^-_{intervalley}$), dark exciton (X^D) and charged biexciton (XX^-) are labelled. **d**, UPL of the high-lying exciton measured with a 600 grooves/mm high-resolution grating showing peaks with 5.8 meV full width at half maximum.

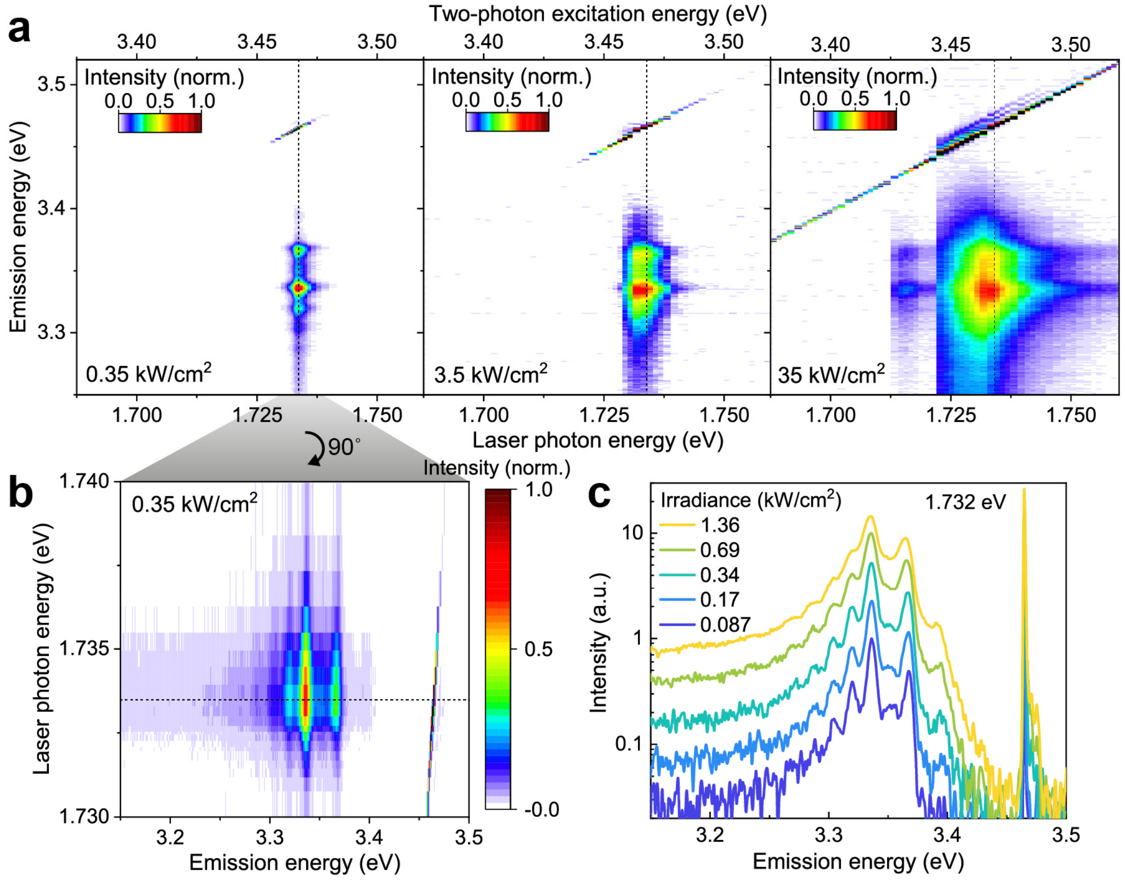

Supplementary Figure 4 | Power dependence of HX UPL and PLE spectra. a, Dependence of the UPL PLE spectrum on pump intensity for irradiances of 0.35 kW/cm^2, 3.5 kW/cm^2 and 35 kW/cm^2. **b**, Rescaled and rotated close-up of the UPL PLE spectrum at a pump irradiance of 0.35 kW/cm^2. **c**, Power dependence of UPL spectra at an excitation photon energy of 1.732 eV. No significant change of the spectral shape of HX emission with excitation intensity is observed.

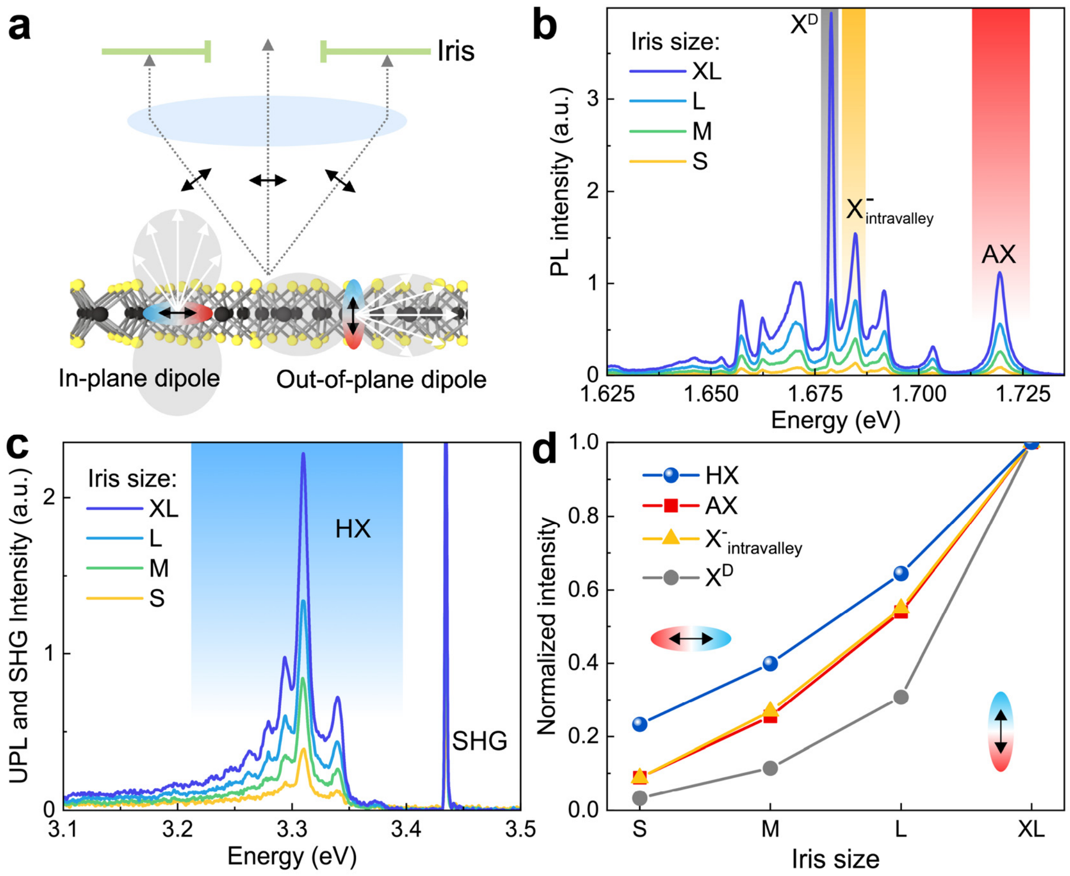

Supplementary Figure 5 | Distinguishing in-plane and out-of-plane dipole orientations of excitons in hBN-encapsulated monolayer WSe_2. Spin-allowed transitions are optically "bright" and have in-plane dipoles, while spin-forbidden transitions are nominally "dark" and only couple with light through out-of-plane transition dipoles[20-22]. **a**, Illustration of the measurement of the dependence of exciton emission on the size of an iris placed in the Fourier plane. A small iris will select in-plane dipoles whereas a larger iris will also allow light from out-of-plane dipoles to pass. (B) Iris size dependence of the PL spectrum of transitions close to the band gap. Four iris sizes were chosen and, for simplicity, are labeled qualitatively as small (S), medium (M), large (L), and extra-large (XL). (C) Dependence of the HX UPL spectrum on iris size. (D) Dependence on iris size of the spectrally integrated excitonic emission (marked red, yellow, grey in (B) and blue in (C)). The dependence is much stronger for the dark exciton X^D than for the neutral A-exciton AX and the trion $X^-_{intravalley}$, since X^D is characterized by an out-of-plane dipole. The dependence on iris size is much weaker for HX than for X^D, implying that HX emits via an in-plane dipole.

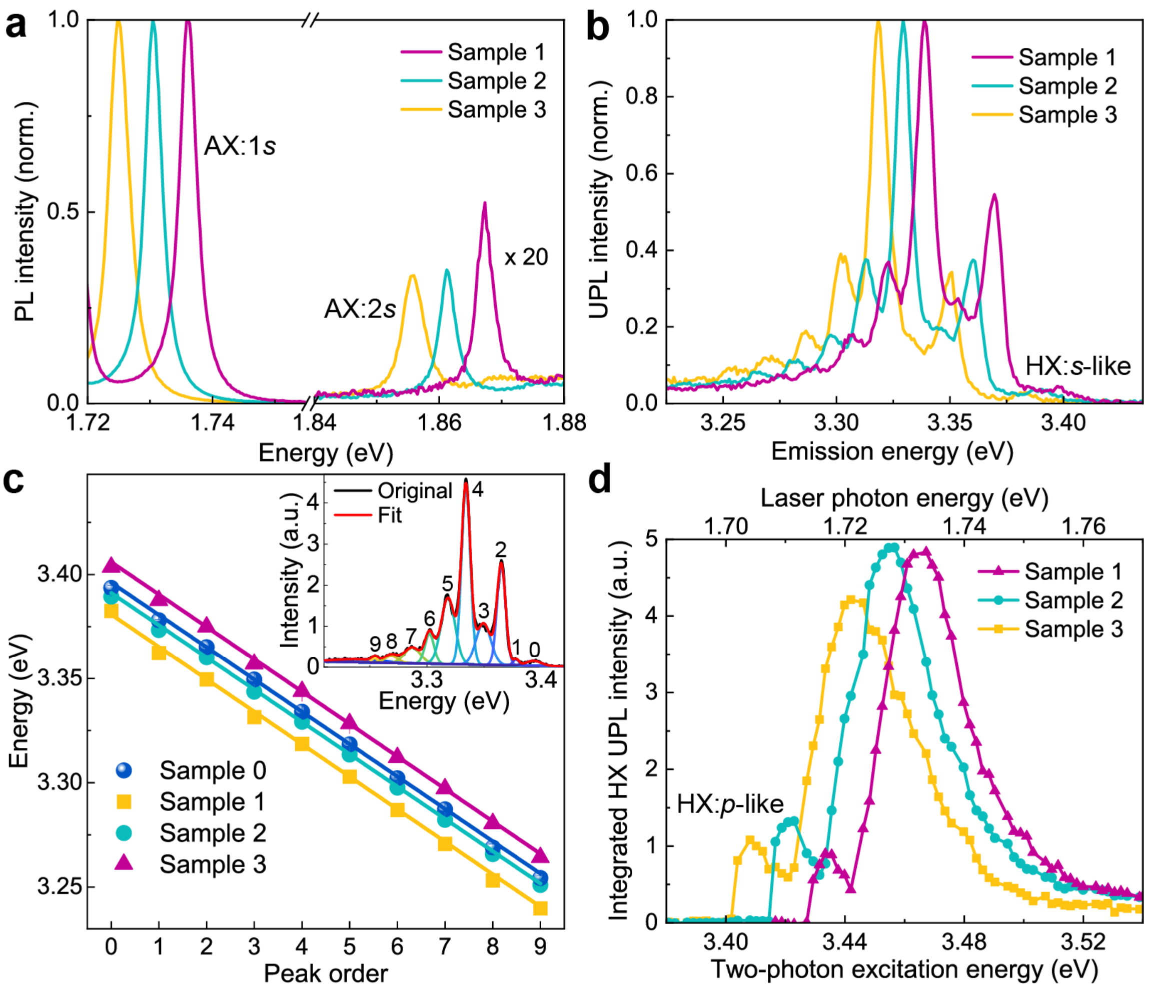

Supplementary Figure 6 | Correlation between the A-exciton (AX) PL and high-lying exciton (HX) PL for different dielectric environments of the WSe_2 monolayer. Samples 1, 2 and 3 correspond to three different WSe_2 monolayers encapsulated in hBN with different thicknesses. **a**, PL of 1*s* and 2*s* states of the A-exciton. **b**, UPL of the high-lying exciton with the lowest-order peak (i.e. peak 0 in Fig. 2 in main text) labelled as the *s*-like HX state. **c**, HX UPL peak positions as a function of peak order obtained through a ten-Gaussian global fit to the three spectra of panel (**b**). The inset shows the fit of Sample 0 in the main text and the result (blue spheres, the same as Fig. 2a) is shown for reference. Linear fits of peak position against peak order determine the spacing of the phonon progression to be 15.50±0.10 meV (Sample 0), 15.59±0.12 meV (Sample 1), 15.50±0.07 meV (Sample 2) and 15.56±0.07 meV (Sample 3), as listed in Supplementary Table 2. **d**, Excitation spectra of the UPL and the two-photon PL at the same irradiance of 35 kW/cm^2, showing the energy of the *p*-like HX state. The lower abscissa indicates double the excitation photon energy. Details of the peak positions are summarized in Table S2.

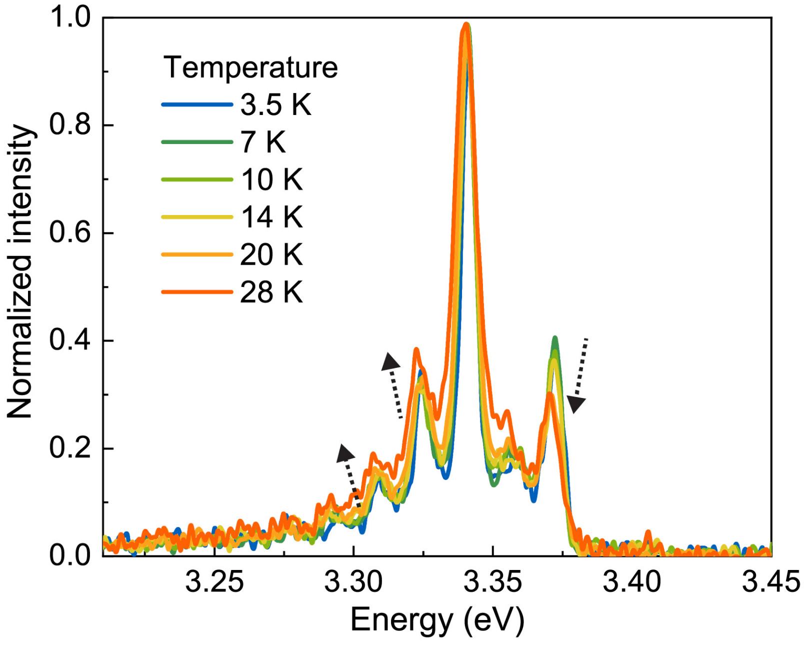

Supplementary Figure 7 | Temperature dependence of HX phonon progression. Normalized UPL as a function of temperature. With temperature increase, the intensities of lower order phonon progression decrease (dashed arrow on the right) relative to the intensities of higher order phonon progression (dashed arrows on the left).

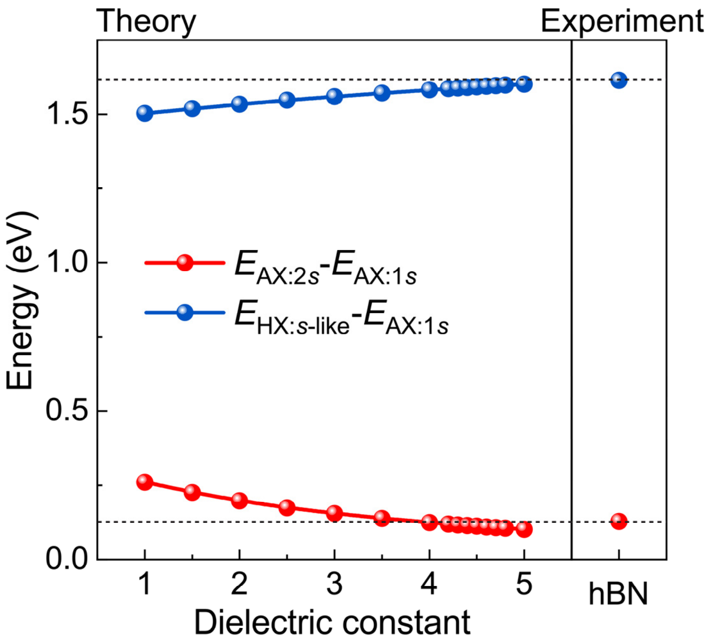

Supplementary Figure 8 | Influence of the dielectric environment on the exciton energy. Rather than evaluating absolute exciton peak energies, we consider the differences in energy among the calculated *s*-like HX, the A:1*s* and the A:2*s* excitons, respectively. The differences are more meaningful since they are more robust against bandgap changes and are nearly independent of *GW* corrections[23]. The left panel shows the calculated dependence of energy differences between A:1*s* and A:2*s*, $E_{AX:2s}$-$E_{AX:1s}$, and between A:1*s* and the zero-phonon line of *s*-like HX, $E_{HX:s\text{-like}}$-$E_{AX:1s}$, on dielectric constant. The calculation is performed within a simple effective-mass model (see Supplementary Methods and Notes). The experimental result for Sample 0 is shown in the right panel. The calculated $E_{HX:s\text{-like}}$-$E_{AX:1s}$ value agrees well with the experimental result, with less than 5% deviation assuming a typical dielectric constant of hBN of 4.5[24,25]. This further supports our interpretation of HX being formed by the negative-mass electron band CB+2- around the *K*-points.

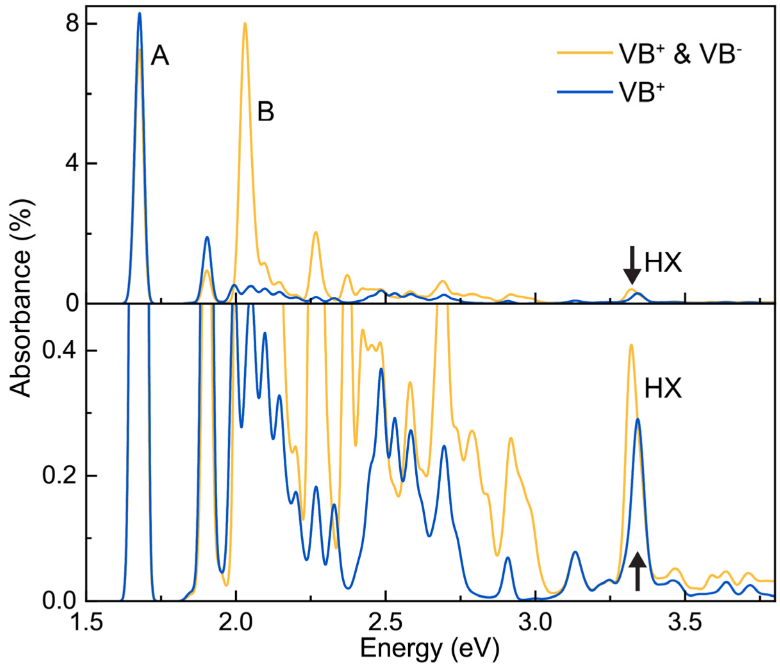

Supplementary Figure 9 | Contributions of the spin-split valence band to exciton formation. Calculated absorbance spectrum of bare monolayer WSe_2 from transitions around the K-points from either VB^+ (blue) or $VB^\pm$ (yellow) to all eight conduction bands $CB^\pm$, $CB+1^\pm$, $CB+2^\pm$ and $CB+3^\pm$. Top panel: plot showing full absorbance spectrum. Bottom panel: close up to show more clearly the absorbance near the HX transition. In addition to the HX from VB^+, a shoulder from VB^- with much weaker oscillator strength overlaps with the HX (black arrow).

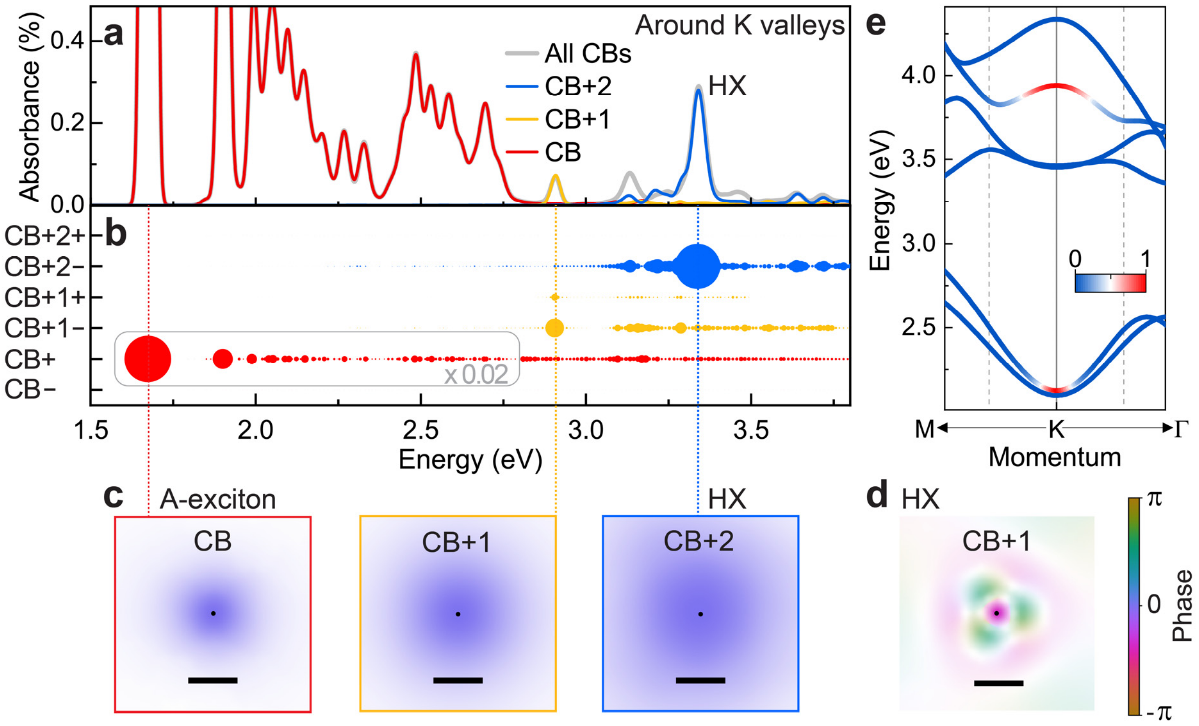

Supplementary Figure 10 | Exciton wavefunction envelopes. a, Calculated absorbance spectrum reproduced from Fig. 4d in main text. **b**, Band contributions to the absorbance spectrum, reproduced from Fig. 4e. **c**, Momentum-space envelope functions of three excitons with dominant contributions from CB (A-exciton), CB+1 and CB+2 (HX). The opacity is proportional to the amplitude of the envelope function and the color denotes the phase according to the scale bar. The black dot marks the *K*-point. The HX evidently has a larger radius in reciprocal space and thus a smaller Bohr radius compared with the A-exciton. The scale bar is 0.2 Å^{-1}. **d**, Even though the HX transition has a dominant contribution from CB+2, the HX also has certain wavefunction amplitudes from CB+1. The figure shows the momentum-space envelope function of the CB+1 contribution to the HX. The wavefunction shows nodes revealing its *p*-like (i.e. odd-parity) non-emissive nature. The contribution of CB+1 to the HX in the absorbance spectrum is therefore minor as seen from the yellow curve in panel b. **e**, Conduction bands of monolayer WSe_2 with the projection of the modulus-squared exciton envelope function of HX onto the CB+2$^-$ band and of AX onto the CB$^-$ band, normalized at the *K*-point. The dashed lines mark the momentum space range used to identify the HX in the calculated absorbance spectrum in (**a**), i.e. within a range of 0.2 Å^{-1} around the *K*-points corresponding overall to 6 % of the total area of the Brillouin zone. The HX is clearly localized around the *K*-points, as seen by the red amplitudes in valence and conduction bands. The effective-mass approximation is therefore still valid.

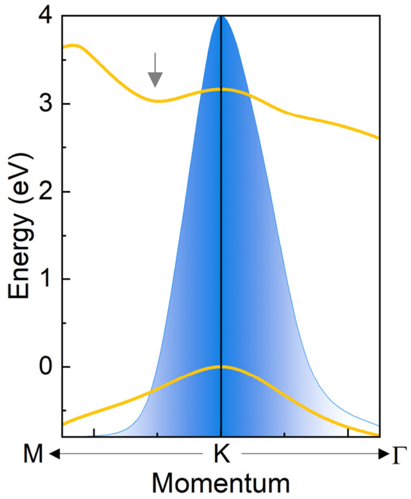

Supplementary Figure 11 | Exciton wavefunction calculated using an effective two-band model. The two bands (yellow curve) are taken from the *ab initio* band structure and the wavefunctions (blue shadow) along the *K*–Γ direction and the *K*–*M* direction are calculated separately as limiting cases to analyze the spreading of the wavefunction. The HX resides around the *K*-points in momentum space instead of spreading out to the local minimum (grey arrow) of the high-lying conduction band, consistent with the full *GW*-BSE calculation.

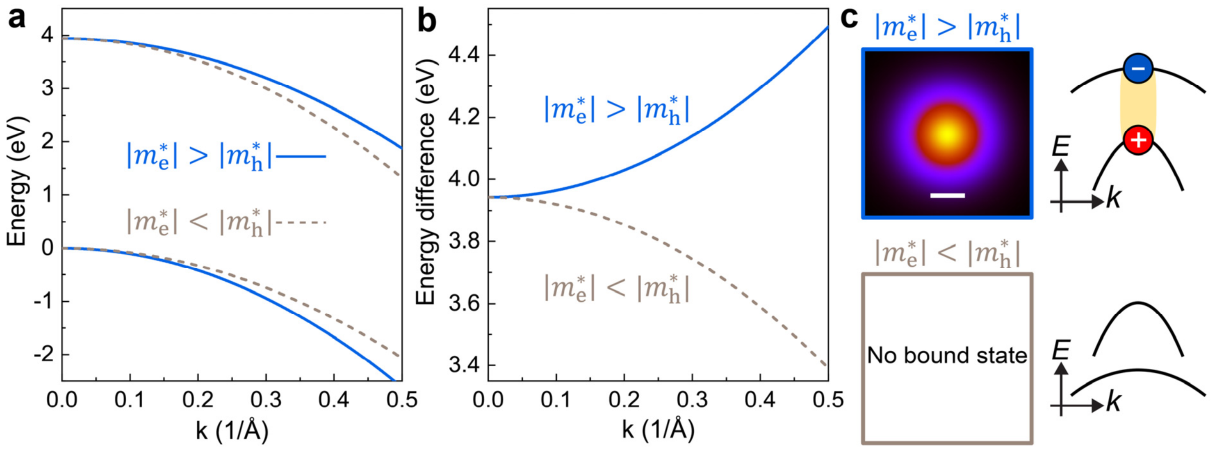

Supplementary Figure 12 | Hydrogen-like model of an exciton using a parabolic band dispersion for the (positive-mass hole) valence band and the (negative-mass electron) conduction band. a, The band dispersions are examined for two different conditions: $|m_e^*| = 0.4604 > |m_h^*| = 0.3636$ (solid blue curves) and $|m_e^*| = 0.3636 < |m_h^*| = 0.4604$ (dashed grey curves). **b**, The corresponding energy difference between conduction and valence bands, $E_e(\vec{k}) - E_h(\vec{k})$, for the two conditions considered. **c**, Probability density $|A(\vec{k})|^2$ of the ground-state exciton wavefunction for the two-mass conditions. The condition of $|m_e^*| > |m_h^*|$ leads to a stable bound exciton localized at $k = 0$ (i.e., in the K-valleys). In contrast, the condition of $|m_e^*| < |m_h^*|$ does not support a bound state at $k = 0$. The scale bar is 0.2 Å^{-1}.

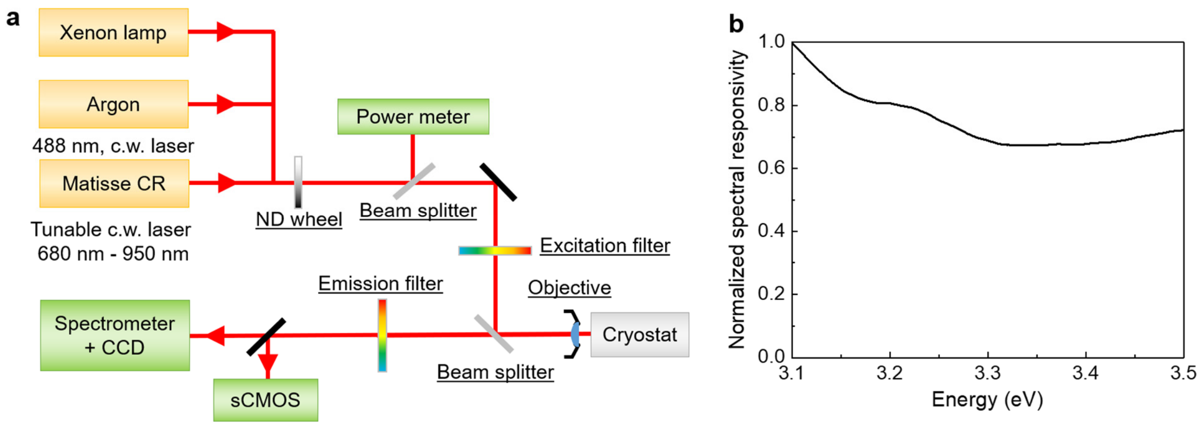

Supplementary Figure 13 | Setup information. a, Illustration of the experimental setup. **b**, Experimentally determined spectral responsivity of the instrument with a calibrated light source.

Excitons	A	B	–	–	HX
Energy (eV)	1.734	2.146	2.513	2.908	> 3.2
Linewidth (meV)	3.02 ± 0.02	61.7 ± 0.6	127 ± 3	172 ± 5	5.8 ± 0.4

Supplementary Table 1 | Energies and linewidths (FWHM) of the excitonic states extracted from the PL and UPL spectra in Fig. 4 and Supplementary Fig. 3.

Sample	AX	HX:*s*-like	HX:*p*-like	$\Delta E_{HX:s\text{-}p}$	ΔE_{phonon}
1	1.7361	3.3980	3.4350	0.0370	0.01559±0.00012
2	1.7305	3.3894	3.4216	0.0322	0.01550±0.00007
3	1.7250	3.3818	3.4091	0.0273	0.01556±0.00007

Supplementary Table 2 | Energies of the excitonic states AX, HX:*s*-like (zero-phonon line) and HX:*p*-like, the energy difference between these states $\Delta E_{HX:s\text{-}p}$, and the phonon progression spacing ΔE_{phonon} from three different samples in Supplementary Fig. 6, in units of eV.

Supplementary References

1 Giannozzi, P. *et al.* QUANTUM ESPRESSO: a modular and open-source software project for quantum simulations of materials. *J. Phys.: Condens. Matter* **21**, 395502 (2009).

2 Perdew, J. P., Burke, K. & Ernzerhof, M. Generalized Gradient Approximation Made Simple. *Phys. Rev. Lett.* **77**, 3865-3868 (1996).

3 Schlipf, M. & Gygi, F. Optimization algorithm for the generation of ONCV pseudopotentials. *Comput. Phys. Commun* **196**, 36-44 (2015).

4 Schutte, W. J., De Boer, J. L. & Jellinek, F. Crystal structures of tungsten disulfide and diselenide. *J. Solid State Chem.* **70**, 207-209 (1987).

5 Hamann, D. R. Optimized norm-conserving Vanderbilt pseudopotentials. *Phys. Rev. B* **88**, 085117 (2013).

6 Hybertsen, M. S. & Louie, S. G. Electron correlation in semiconductors and insulators: Band gaps and quasiparticle energies. *Phys. Rev. B* **34**, 5390-5413 (1986).

7 Rohlfing, M. & Louie, S. G. Electron-hole excitations and optical spectra from first principles. *Phys. Rev. B* **62**, 4927-4944 (2000).

8 Deslippe, J. *et al.* BerkeleyGW: A massively parallel computer package for the calculation of the quasiparticle and optical properties of materials and nanostructures. *Comput. Phys. Commun* **183**, 1269-1289 (2012).

9 Ismail-Beigi, S. Truncation of periodic image interactions for confined systems. *Phys. Rev. B* **73**, 233103 (2006).

10 da Jornada, F. H., Qiu, D. Y. & Louie, S. G. Nonuniform sampling schemes of the Brillouin zone for many-electron perturbation-theory calculations in reduced dimensionality. *Phys. Rev. B* **95**, 035109 (2017).

11 Zollner, K., Faria Junior, P. E. & Fabian, J. Giant proximity exchange and valley splitting in transition metal dichalcogenide/hBN/(Co, Ni) heterostructures. *Phys. Rev. B* **101**, 085112 (2020).

12 Zollner, K., Faria Junior, P. E. & Fabian, J. Proximity exchange effects in MoSe2 and WSe2 heterostructures with CrI3: Twist angle, layer, and gate dependence. *Phys. Rev. B* **100**, 085128 (2019).

13 Rytova, N. S. The screened potential of a point charge in a thin film. *Mosc. Univ. Phys. Bull.* **3**, 18 (1967).

14 Keldysh, L. V. Coulomb interaction in thin semiconductor and semimetal films. *Sov. J. Exp. Ther. Phys. Lett.* **29**, 658 (1979).

15 Cudazzo, P., Tokatly, I. V. & Rubio, A. Dielectric screening in two-dimensional insulators: Implications for excitonic and impurity states in graphane. *Phys. Rev. B* **84**, 085406 (2011).

16 Kresse, G. & Furthmüller, J. Efficient iterative schemes for ab initio total-energy calculations using a plane-wave basis set. *Phys. Rev. B* **54**, 11169-11186 (1996).

17 Togo, A. & Tanaka, I. First principles phonon calculations in materials science. *Scr. Mater.* **108**, 1-5 (2015).

18 Monserrat, B. Electron–phonon coupling from finite differences. *J. Phys.: Condens. Matter* **30**, 083001 (2018).

19 Lin, K.-Q., Bange, S. & Lupton, J. M. Quantum interference in second-harmonic generation from monolayer WSe2. *Nat. Phys.* **15**, 242-246 (2019).

20 Wang, G. *et al.* In-plane propagation of light in transition metal dichalcogenide monolayers: optical selection rules. *Phys. Rev. Lett.* **119**, 047401 (2017).

21 Zhou, Y. *et al.* Probing dark excitons in atomically thin semiconductors via near-field coupling to surface plasmon polaritons. *Nat. Nanotechnol.* **12**, 856 (2017).
22 Zhang, X.-X. *et al.* Magnetic brightening and control of dark excitons in monolayer WSe2. *Nat. Nanotechnol.* **12**, 883-888 (2017).
23 Waldecker, L. *et al.* Rigid Band Shifts in Two-Dimensional Semiconductors through External Dielectric Screening. *Phys. Rev. Lett.* **123**, 206403 (2019).
24 Geick, R., Perry, C. H. & Rupprecht, G. Normal Modes in Hexagonal Boron Nitride. *Phys. Rev.* **146**, 543-547 (1966).
25 Stier, A. V. *et al.* Magnetooptics of Exciton Rydberg States in a Monolayer Semiconductor. *Phys. Rev. Lett.* **120**, 057405 (2018).
